# Supplementary material for: BZR1 Physically Interacts with SPL9 to Regulate the Vegetative Phase Change and Cell Elongation in Arabidopsis
Source: Int J Mol Sci. 2021 Sep 27;22(19):10415. doi: 10.3390/ijms221910415 (PMC8509050; doi:10.3390/ijms221910415)
Supplement: Supplementary file 1 [file ijms-22-10415-s001.zip › ijms-1338428-supplementary.pdf]

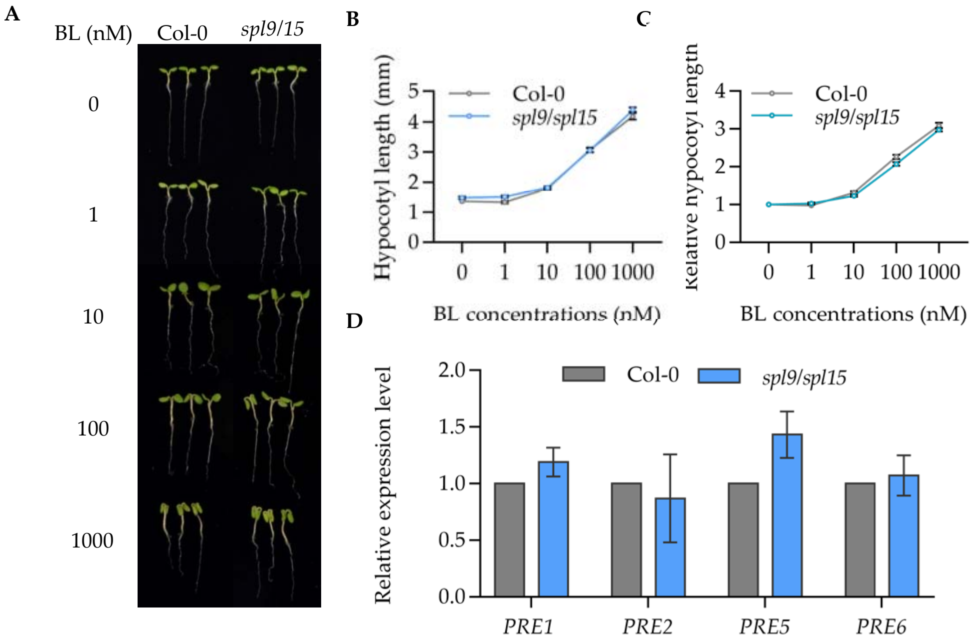

Figure S1. **The double mutant of spl9/spl15 displayed same BR sensitivity on hypocotyl elongation.**

(A) Representative images of seven-day-old seedlings of wild-type, and *spl9/spl15* grown on the 1/2 MS medium with different concentrations of BL. (B) and (C) The hypocotyl lengths were measured from at least 30 plants. Relative hypocotyl length were average of 30 plants and normalized to the untreated plants. Error bars represent standard deviation. (D) Quantitative RT-PCR analysis the expression of members of PRE family in wild type, *spl9* and *spl9/spl15*. Seedlings of wild type and indicated plants were grown on ½ MS medium under constant light for 7 days. *PP2A* gene was analyzed as an internal control. Error bars represent standard deviation of three independent experiments.

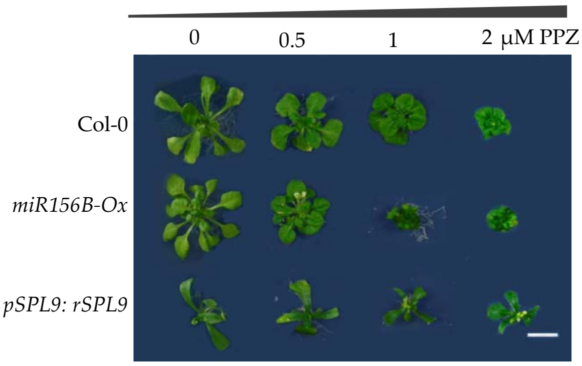

Figure S2. The growth phenotype of wild type, *miR156-Ox* and *pSPL9:rSPL9* in the presence of PPZ.

Seedlings of Col-0, *pSPL9:rSPL9* and *miR156-Ox* were grown on 1/2 MS medium with different concentrations of PPZ under long day condition for 4 weeks.

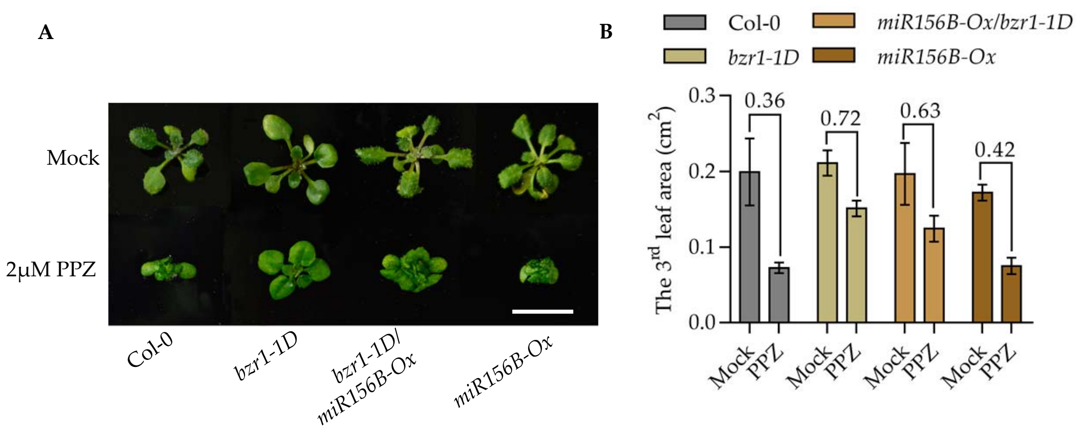

Figure S3. ***MiR156*** **overexpression partially suppressed the PPZ resistance of** ***bzr1-1D*****.**

(A) The representative image of 3-week-old wild type, *miR156B-Ox, bzr1-1D* and *bzr1-1D/miR156B-Ox* plants. **(B)** The leaf area of 3rd rosette leaf of wild type and indicated plants. Error bars represent standard deviation (n=10). The numbers above the column indicated leaf area ratio of PPZ treatment to mock.

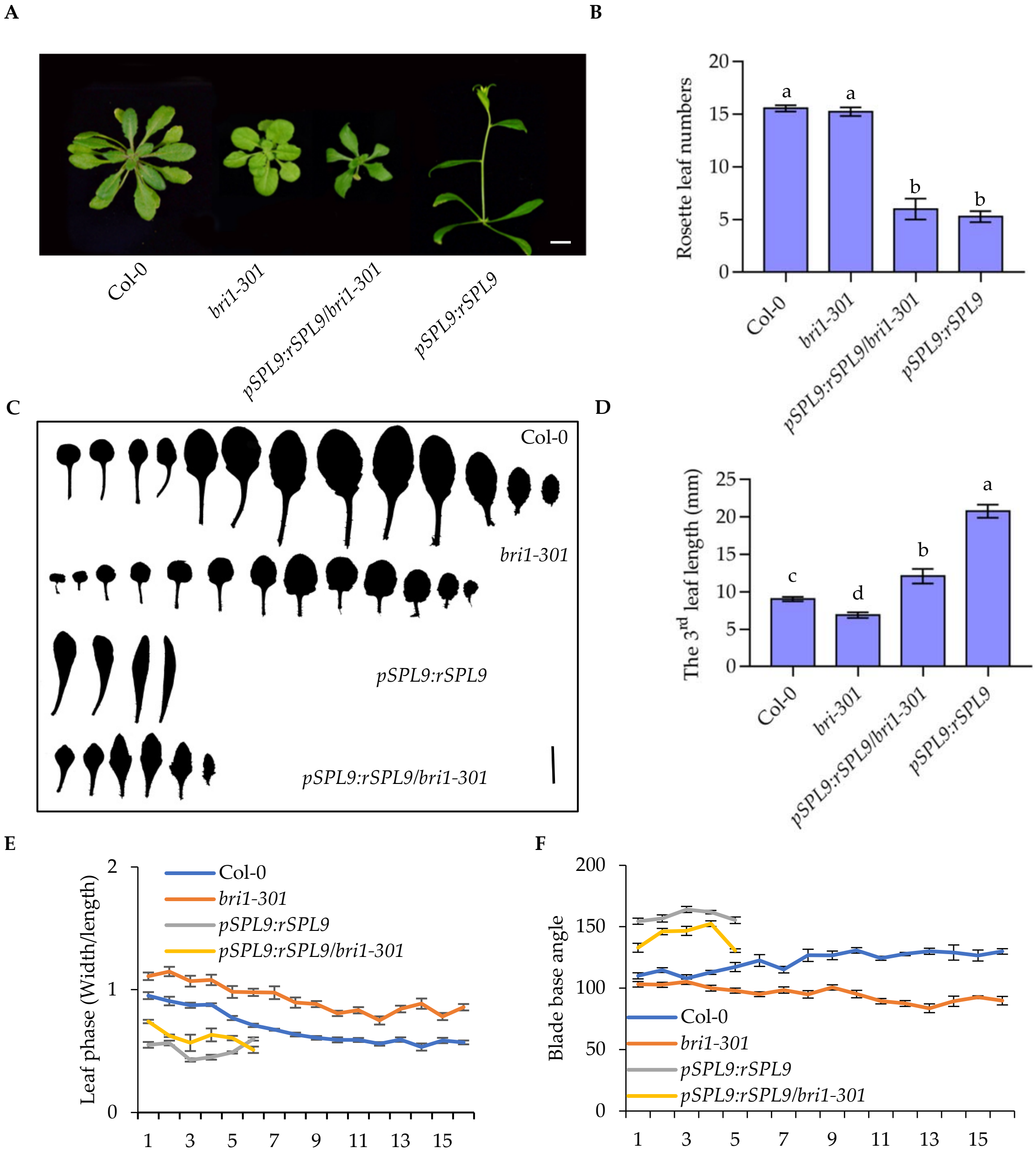
A
B
Col-0
bri1-301
pSPL9:rSPL9/bri1-301
pSPL9:rSPL9
Rosette leaf numbers
20
15
10
5
0
a
a
b
b
C
D
Col-0
bri1-301
pSPL9:rSPL9
pSPL9:rSPL9/bri1-301
The 3rd leaf length (mm)
25
20
15
10
5
0
c
d
b
a
Col-0
bri-301
pSPL9:rSPL9/bri1-301
pSPL9:rSPL9
E
F
Leaf phase (Width/length)
2
1
0
1
3
5
7
9
11
13
15
Col-0
bri1-301
pSPL9:rSPL9
pSPL9:rSPL9/bri1-301
Blade base angle
200
150
100
50
0
1
3
5
7
9
11
13
15
Col-0
bri1-301
pSPL9:rSPL9
pSPL9:rSPL9/bri1-301

Figure S4. The delayed vegetative phase change of bri1-301 was suppressed by *pSPL9:rSPL9* plants.

(A) The representative image of 5-week-old wild type, *bri1-301*, *pSPL9:rSPL9* and *pSPL9:rSPL9/bri1-301* plants. **(B)** The rosette leaf numbers of wild type and indicated plants after flowering. Error bars represent standard deviation (n=10). Different letters above the bars indicated statistically significant differences between the samples (ANOVA analysis followed by Uncorrected Fisher's LSD multiple comparisons test, $p < 0.05$). **(C)** The shape phenotypes of full expanded rosette leave of wild type and indicated plants. **(D)** The 3rd leaf length of wild type and indicated plants after initiation different days. The leaf lengths were measure from at least 30 plants. Error bars represent standard deviation. **(E)** The ratio of leaf width to leaf length of full expanded rosette leaves of wild type and indicated plants. The leaf width and leaf length were measure from at least 30 plants. Error bars represent standard deviation. **(F)** The base angle of full expanded rosette leaves of wild type and indicated plants. The leaf base angle was measure from at least 30 plants. Error bars represent standard deviation.

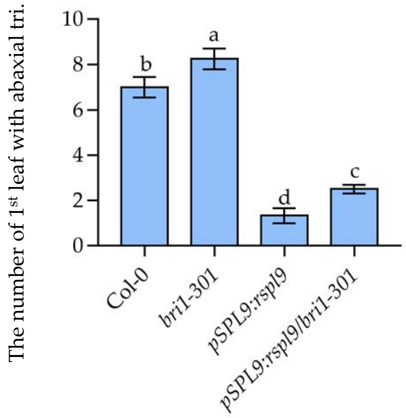

Figure S5. *bri1-301* can partly rescue the less abaxial trichome phenotype of *pSPL9:rSPL9*.

The growth phenotype of 5-week-old wild type, *bri1-301*, *pSPL9:rSPL9* and *pSPL9:rSPL9/ bri1-301* plants. The number of 1st leaf with abaxial trichome of indicated plant were calculated from at least 10 plants. Error bars represent standard deviation.
