# Supplementary material for: BZR1 Physically Interacts with SPL9 to Regulate the Vegetative Phase Change and Cell Elongation in Arabidopsis
Source: Int J Mol Sci. 2021 Sep 27;22(19):10415. doi: 10.3390/ijms221910415 (PMC8509050; doi:10.3390/ijms221910415)
Supplement: Supplementary file 1 [file ijms-22-10415-s001.zip › Table S1.pdf]

Table S1. Oligonucleotide sequences used in this paper.

Gene cloning

Gene	Forward	Reverse
SPL9	ATGGAGATGGGTTCCAACTCG	GAGAGACCAGTTGGTATGG
BIN2	ATGGCTGATGATAAGGAGATG	AGTTCCAGATTGATTCAAGAAGC
BZR1	ATGACTTCGGAGCTACG	ACCACGAGCCTTCCCATTTCC

Mutant Genotyping

Gene	Forward	Reverse
DET2 (*det2-1*)	AGGAGGCTGGTTCGAGTTGGTA	AAGGAATAACAGCTTTACGAGT
BRI1 (*bri1-301*)	ATGAAGACTTTTTCAAGCTTCTTTC	TTTTCCTTCAGGAACTTCTTTTATAC
BIN2 (*bin2-1*)	CACAATGTTGCTGGAGTTGCTCACAGAGATCTA	AGTTCCAGATTGATTCAAGAAGC

qPT-PCR

Gene	Forward	Reverse
PP2ART	TTCTCGCTCCAGTAATGGGATCCGA	GTTCTCCACAACCGCTTGGTCGACT
SPL9RT	TGTGGCTGGTATCGAACAGAGG	TTCCGGAAGCTGATGAAACCTG
SPL10RT	GCGTTTCAAACATCTGGTGGGTTC	CCTGAGCATTCTCCCACACCTTTG
SPL15RT	TCGCTCCATCTCTTTACGGAAACC	TGCATCACTGATCTTGCGGTTG
PRE1RT	GTTCTGATAAGGCATCAGCCTCG	CATGAGTAGGCTTCTAATAACGG
PRE2RT	ACTCTGTGTGGTGGCACAACTG	ACGTTGACTCCAAGGACCACTC
PRE5RT	AACGGCGTCGTTCTGATAAG	CATGAGTAAGCTTCTAATCACGG
PRE6RT	ACCGTTTGTCGGAACTCTTGGC	AGCTCCTAATGATGGCTGCTTCG
